# Clinical Case of Immune Dysregulation, Polyendocrinopaty, Enteropathy, X-Linked (IPEX) Syndrome with Severe Immune Deficiency and Late Onset of Endocrinopathy and Enteropathy

**DOI:** 10.1155/2014/564926

**Published:** 2014-06-01

**Authors:** R. Savova, M. Arshinkova, J. Houghton, M. Konstantinova, M. Gaydarova, E. Georgieva, G. Popova, V. Genova, N. Tomova, A. Anadoliiska, K. Koprivarova

**Affiliations:** ^1^University Pediatric Hospital, Sofia, Bulgaria; ^2^Medical School, University of Exeter, UK

## Abstract

*Objective*. To describe the clinical characteristics of IPEX syndrome in a child with *FOXP3* mutation. *Clinical Case*. A boy aged 2.3 years was born from first normal pregnancy with a weight of 3420 gr. *Family History*. Two brothers of the mother died before the age of 3 years with severe infections, diarrhea, erythroderma, and elevated immunoglobulins class E (IgEs). Since first month of life, our patient suffered from septicemia, pneumonias, pyelonephritis, and meningitis, accompanied with eczematous dermatitis and IgEs up to 4000 IU/L (normal <10). At the age of 1.6 years, he developed type 1 diabetes mellitus (T1DM). He was underweighted (−3.42 SDS) and had some phenotypic features like coarse face, muscle hypotonia, joint hyperextensibility, eczematous dermatitis, and subcutaneous cold abscesses. Autoimmune thyroiditis and celiac disease were excluded. After diabetes, intermittent watery diarrhea appeared with progression to severe intractable form. Finally, aggravating symptoms of nephritis, cachexia, and respiratory insufficiency were the cause for his death at the age of 2 years and 3 months. The DNA analysis at the University of Exeter Medical School established mutation at exon 10 of *FOXP3* gene c.1010G >A, p. (Arg337Gln), which confirmed IPEX syndrome. The same mutation in heterozygotic state was found in the mother. A prenatal diagnosis of her second pregnancy ensured a daughter carrier of the mutation.

## 1. Introduction

Type 1 diabetes mellitus (T1DM) is an autoimmune disease which incidence rises worldwide being triggered by unknown yet environmental factors. The etiology is considered to be multifactorial. The major histocompatibility complex (MHC) on chromosome 6p21.3 is the main susceptibility locus for many autoimmune diseases [1] and for type 1 DM as well [2]. The second most important is insulin gene* (INS)* on chromosome 11p15 [2]. Genome-wide linkage analysis for T1DM-susceptibility loci supports an interaction between HLA and more than 40 non-HLA loci and evaluated a series of non-HLA genes responsible for immune regulation:* CTLA-4* on 2q33.2 and 17p13.1,* PTPN22* on 1p13.2, interleukin- (IL-) 2 receptor *α* chain* (IL2RA)* on 10p15,* IFIH1* on 2q24.2,* IL10, IL19,* and* IL20* on 1q32.1,* CD69* on 12p13.31,* IL27* on 16p11.2, and* PTPN2* on 18p11.21 [3–5]. T1DM is often present in autoimmune polyendocrine syndrome type 1 (APS1) and type 2 (APS2) and IPEX syndrome, reaching 20% in APS1, 50% in APS2, and >60% in IPEX [6]. These syndromes are caused by immunologically mediated destructive process conducted by autoreactive T cells, which damage multiple endocrine organs and nonendocrine tissues due to compromised central (APS1) or peripheral (APS2) immune tolerance. In patients with primary immune deficiency disorders (PIDDs), a breakdown of self-tolerance mechanisms often exists with high incidence of autoimmunity. FOXP3 functions as a master of transcription for the development of regulatory T cells (T-reg) both in human and in mice. In the patients with IPEX syndrome, the lack of the forkhead box protein 3 in lymphoid tissues (thymus, spleen, and lymph nodes), encoded by* FOXP3* gene at Xp11.23-Xq13.3, leads to impaired development and suppressed function of CD4+CD25+ *T*-reg. It causes dysfunction of the effector T cells (T-eff) and predisposes to multiple autoimmune diseases as well. The protein, encoded by* FOXP3,* has 4 functional domains in its molecular structure: (1) N-terminal proline-rich repressor domain; (2) zinc-finger domain; (3) leucine-zipper domain; (4) and DNA-binding forkhead/winged helix domain (FKH) [7]. Mutations that affect any of these key functional domains may alter or abrogate the ability of the transcription factor to regulate gene expression in T-reg. Recent studies showed that mature naïve B cells from IPEX patients often expressed autoreactive antibodies, suggesting an important role for T-reg in maintaining peripheral B cell tolerance [8] and B cell anergy [9]. IPEX syndrome is an X-linked genetic disorder in which T1DM is a common feature and can be seen as early as in the 1st month of life. In the cohort of persistent genetically inherited neonatal diabetes, IPEX-related diabetes is unique because of its autoimmune nature. It was found in 4% of neonatal permanent diabetes among males [10]. Other symptoms due to profound immune dysregulation that can be seen in males with IPEX syndrome include enteropathy (100%), dermatitis (65%), failure to thrive (50%), thyroiditis (30%), and recurrent infections (20%). Many other autoimmune phenomena like nephritis, pneumonitis, hepatitis, vasculitis, arthritis, myositis, alopecia, and autoimmune cytopenias can be presented [11]. Severe watery diarrhea and life-threatening infections shorten the lifespan of these patients and they usually die in the first 1-2 years of life.

The aim of our paper is to present a boy with IPEX syndrome and to highlight the main clinical characteristics and outcome of this severe disorder. The boy was aged 2 years and 3 months at the time of the last hospitalization. Family history is as follows: healthy parents, but two brothers of the mother died at age of 7 months and 3 years with severe infections, chronic diarrhea, and skin eruptions reported as eczematous dermatitis/erythroderma. In one of them, highly elevated IgEs were measured and Job syndrome was suspected. The boy presented here was born after first normal pregnancy by Cesarean section with a weight of 3420 g and length of 51 cm. Oxygen therapy was provided 10 days after delivery. At the age of 1 month, he was hospitalized for pneumonia and septicemia due to* Streptococcus pneumoniae*. Skin eruptions assessed as erythroderma were present. Humoral immunity was evaluated and elevated IgEs 855 IU/mL were found. Positive specific IgEs toward cow's milk proteins were established and feeding with dietary hypoallergic formula was introduced. In early infancy, the child suffered from multiple severe bacterial infections, pruritic eczematous dermatitis, elevated IgEs, and eosinophilia. Hypotrophy, subcutaneous cold abscesses, and enlarged peripheral lymph nodes appeared over time together with other phenotypic features resembling Job syndrome like coarse face and joint hyperextensibility. At the age of 1 year and 6 months, autoimmune diabetes mellitus was diagnosed. After diabetes, watery diarrhea appeared, being intermittent initially, but persisting during the second year of age and leading to severe malnutrition (Table 1).

At the last hospitalization, the main clinical characteristics were severe malnutrition, cachexy, and fluid retention due to hypoproteinemia. A complex treatment was provided with total parenteral nutrition, drainage of peritoneal transudation, antibiotics, and diuretics. Insulin reduction to 0.3 U/kg b.w. did not lead to ketoacidosis. One week later, a new symptom was tachypnea with bilateral crepitate of the lung without initial pathologic changes on the first X-ray examination. Blood oxygen saturation fell down and oxygen supply was started. The child was highly oxygen dependent and cyanosis appeared minutes after discontinuation of the oxygen. Corticosteroid treatment with methylprednisolone i.v. at a daily dose of 3 mg/kg b.w. was the last attempt to help the child. Some minimal clinical benefit with reduced watery stools and better tolerance to oral feeding was observed, but severe respiratory insufficiency persisted and was the cause of the death. The bronchial smear for* Pneumocystis carinii* could not be investigated. The parents did not agree on autopsy and some specific organ damages remained obscure.

After the diagnosis of diabetes, DNA samples of the child and the parents were sent to the University of Exeter Medical School. The DNA analysis found mutation at exon 10 of* FOXP3 *gene c.1010G > A, p. (Arg337Gln), which confirmed IPEX syndrome. The same mutation in heterozygotic state was found in the mother. A prenatal diagnosis of her second pregnancy was performed and a daughter carrier of the mutation was born.

## 2. Discussion

If a patient has immune deficiency and hyper-IgE syndrome without the classical triad of watery diarrhea, dermatitis, and T1DM/autoimmune thyroiditis in the first months of life, pediatricians should consider several primary immune deficiency disorders with hyper-IgE syndrome: hyper-IgE syndrome (HIES) or Job syndrome, Wiskott-Aldrich syndrome, IPEX, Omenn syndrome, and atypical complete DiGeorge syndrome [12].

Some phenotype characteristics of our patient like coarse face, hyperextensible joints, eczematous dermatitis, and clinical history of severe recurrent infections with hyper-IgE syndrome resembled Job syndrome and this diagnosis was considered before the development of watery diarrhea and autoimmune diabetes. HIES is now recognized as a primary immunodeficiency disease characterized by recurrent skin abscesses, cold abscesses, recurrent pneumonia with pneumotocele, eczematous dermatitis, and elevated serum IgEs together with nonimmune features like coarse face, joint hyperextensibility, and skeletal/dental abnormalities. HIES was initially reported to have an autosomal dominant (AD) inheritance pattern, but cases with autosomal recessive (AR) inheritance and sporadic ones have been reported. AR HIES differs from AD HIES with lack of skeletal or dental anomalies and pnematocele development, but with susceptibility to viral infection including* Molluscum contagiosum* and neurological complications for unknown reasons [13]. Mutations of the signal transducer and activator of transcription 3 (*STAT3*) gene on 17q21.2 and deficiency of* DOCK8* (dedicator of cytokinesis 8) on 9p24.3 are now known to cause a combined immunodeficiency rendering most of the affected patients susceptible to viral, fungal, and bacterial infections.

In our patient with primary immune deficiency with hyper-IgE, the diagnosis of IPEX syndrome was considered after diabetes and watery diarrhea appeared during the second year of life. The mutation found R337Q corresponded with more severe and classical clinical presentation of the disorder [10] with enteropathy, dermatitis, and autoimmune diabetes. This mutation is located at FKH domain, which spans over amino acids 337–421 in the C-terminal region of the protein. Like the N-terminal region, FKH is necessary for most aspects of* FOXP3 *function [7]. In typical cases, severe enteropathy and diabetes developed as early as the first month of life. In some patients, an intrauterine hypotrophy and pancreatic hypoplasia showed very early autoimmune process [10]. Hyper-IgE, eczematous dermatitis, and immune deficiency with recurrent infections and cold abscesses typical not only for Job syndrome but also for IPEX syndrome were present in our patient. We can speculate about the presence of aggravating autoimmune nephritis and pneumonitis but organ biopsy could not be performed because of the critical condition of our patient. Other genes possibly related to severe immune deficiency were not investigated. The same* FOXP3* mutation was suspected in both brothers of the mother who died in early infancy with immune deficiency and hyper-IgE.

X-linked syndrome with diarrhea, polyendocrinopathy, and fatal infections in infancy was reported for the first time by Powell et al., in 1982 [14]. Later, Shigeoka et al. reported that a novel X-linked immunodeficient disease with autoimmunity and enteropathy had a locus mapped next to the Wiskott-Aldrich locus Xp11.2 [15]. Ferguson et al. performed linkage analysis on 20 members of affecter kindred mapping the locus to the pericentromeric region Xp11.23-q21.1 [16] and concluded that this was an X-linked disorder, distinct from Wiscott-Aldrich syndrome. By linkage analysis in a large pedigree, Bennet et al. mapped the locus for X-linked polyendocrinopathy, immune dysfunction, and diarrhea (IPEX) to a 17 cM interval defined by markers DXS8083 and DXS8107 at Xp11.23-q13.3 [17]. Torgerson, 2007, investigated a possible phenotype-genotype correlation in more than 100 symptomatic patients from more than 50 families. In the patients with a clinical phenotype compatible with IPEX, approximately 50% were found to have mutations in* FOXP3.* These include missense, splice site, and deletion mutations [18]. After adding new genetically proven cases to the cohort of IPEX syndrome over the world, considerable clinical heterogeneity was established. Some mutations of* FOXP3 *lead to functionally hypomorphic proteins that presented milder clinical features of the syndrome and partially suppressed T-reg [19]. Some mutations that do not abrogate the forkhead domain were found in 7-year-old boy and unrelated 24-year-old men with autoimmune enteropathy not associated with T1 DM or other endocrinopathies [20]. A minimal change nephrotic syndrome together with classical presentation of IPEX was reported by Hashimura et al. [21]. Neonatal diabetes and nephrotic syndrome were reported as the only clinical features in the surviving 15-year-old patient with mutation V408M of* FOXP3 *[10]. IPEX was genetically proven in 11-year-old boy with diabetes, pure red cell aplasia, membranous glomerulopathy, and posterior reversible encephalopathy syndrome after a vaccination against influenza virus [22]. These atypical clinical presentations of IPEX indicated existing possible underestimation of the real morbidity. Similar genotypes did not always result in similar phenotypes in terms of disease presentation and severity of IPEX [23]. Several epigenetic regulations of* FOXP3* expression like methylation of CpG residues were reviewed by Lal and Bromberg [24]. A highly conserved CpG enriched element located at 5′ UTR of* FOXP3* was identified as the T-reg specific demethylated region (TSDR) [25]. Demethylated CpG supports full expression of* FOXP3*. It is constantly demethylated exclusively in T-reg but not in T-eff, where it is fully methylated. Unlike fluocytometry demethylation status of TSDR could differentiate peripheral T-reg from T-eff, which are* CD25 *and* FOXP3* positive when activated in a state of inflammation. After analyzing demethylation status of TSDR of* FOXP3* within peripheral CD3+T cells of 28 IPEX-like patients (including females) without* FOXP3* mutation, Barzaghi et al. [26] showed significantly reduced amount of peripheral T-reg, compared to both healthy subjects and unrelated disease controls.

Etiology of immune phenomena in other IPEX-like patients needs to be established in the future.

## 3. Conclusion

We present a classical case of IPEX syndrome in a boy with severe immune deficiency, hyper-IgE, and eczematous dermatitis in the first year of life and late onset endocrinopathy and intractable watery diarrhea during second year. The identification of a* FOXP3* mutation in this family was important to predict prognosis for the child and risk for future offspring and enabled prenatal genetic diagnosis.

## Figures and Tables

**Table 1:** Disease history with main symptoms, somatic features, and laboratory data.

Age	Weight/height SDS	Main diagnosis and symptoms. Treatment	Somatic features	Laboratory features
1 month	N/A	Septicemia, pneumonia	Erythroderma	IgE 855 IU/mL (<10)

3–5 months	N/A	Pneumonias Urinary tract infections Intermittent dyspepsia Meningitis	Hypotrophy Enlarged peripheral lymph nodes Pruritic eczematous dermatitis	N/A

6 months	Weight 4590 g (−3.67 SDS) Height 60 cm (−2.6 SDS)	Bronchiolitis Complex treatment with respiratory therapy, antibiotics, topical emollients/steroids, antihistamines, and dietary feeding with protein hydrolyzed lactose free formula	Hypotrophy Enlarged peripheral lymph nodes Pruritic eczematous dermatitis Muscle hypotonia Left kidney malrotation Hydronephrosis and mild vesicoureteral reflux	IgG 9.11 g/L (1.39–9.34) IgM 1.02 g/L (0.2–1.2) IgA 0.38 g/L (0.04–0.78) IgE > 4000 IU/mL (<10) Eosinophilia 13% Normal T and B cell subset quantification Mild iron deficiency Hemoglobin 115 g/L

1.6 years	7400 g (−3.42 SDS)	Diabetes mellitus Right thigh phlegmon Transient watery diarrhea with weight loss up to 6800 g Insulin dose of 1.3 U/kg b.w. Surgical incision and antibiotics Protein hydrolyzed lactose free formula and gluten-free diet	Coarse face with prominent forehead, broad nasal bridge, bulbous nose, and macroglossia Joint hyperextensibility Subcutaneous cold abscesses on the neck and face Eczematous dermatitis Napkin's dermatitis Balanoposthitis	Blood sugar 27 mmol/L No ketones Anti-GAD65 antibodies 32 ng/mL (normal <32) Anti-insulin antibodies 60.1 IU/mL (<10) Basal C-peptide 158.7 pmol/L (198–960) Tissue transglutaminase IgA antibodies 5.2 U/mL (<7.0) TSH 1.34 mIU/L (0.4–4.2) fT4 11.2 pmol/L (9.8–18.9)

1.9 years	9300 g (−2.80 SDS)	Intermittent watery diarrhea Insulin dose 0.75 U/kg b.w. Lactose/gluten-free diet	Mentioned above	HbA1c 7.65% (4–6) (COBAS INTEGRA/Roche diagnostic system)

2.1–2.3 years	7000–11500 g	Severe watery diarrhea Hypoglycemias Vomiting and intolerance to oral feeding Reduced insulin dose 0.3 to 0.1 U/kg b.w Parenteral nutrition Antibiotics i.v. Corticosteroids i.v. Drainage of peritoneal transudation Diuretics	Mentioned above + severe malnutrition Enlarged tensed abdomen, visible abdominal wall veins, intestinal sacculations, and significant abdominal dropsy Lung hypervolemia on X-ray examination	Proteinuria with granular and hyaline casts Hypoproteinemia 52.8 g/L Na 120 mmol/L Ca 1.89 mmol/L K 3.9 mmol/L Metabolic nonketotic acidosis (pH 7.15) Normal urea Creatinine and transaminases Uroculture negative Varied bacterial and *Candida* contamination of the stools
